# Increased severity of two concurrent *Campylobacter jejuni* clones causing large outbreaks, Denmark, July to October 2025

**DOI:** 10.2807/1560-7917.ES.2025.30.47.2500856

**Published:** 2025-11-27

**Authors:** Guido Benedetti, Gitte Sørensen, Ana Paula Finatto Canabarro, Emily Dibba White, Tine Graakjær Larsen, Susanne Schjørring, Eva Møller Nielsen, Luise Müller, Steen Ethelberg, Katrine Grimstrup Joensen

**Affiliations:** 1 Department of Infectious Disease Epidemiology and Prevention, Statens Serum Institut, Copenhagen, Denmark; 2 Department of Bacteria, Parasites and Fungi, Statens Serum Institut, Copenhagen, Denmark; 3 ECDC Fellowship Programme, Field Epidemiology path (EPIET), European Centre for Disease Prevention and Control (ECDC), Stockholm, Sweden; 4 Department of Public Health, Global Health Section, University of Copenhagen, Copenhagen, Denmark

**Keywords:** Campylobacter jejuni, outbreak, whole genome sequencing, hospitalisation, poultry, food safety regulation

## Abstract

In July–October 2025, two concurrent *Campylobacter jejuni* outbreaks (clones ST49#3 and ST52#4) with 112 notified cases were detected through Denmark’s whole genome sequencing surveillance programme. The outbreaks were clinically severe: 45 (40%) infected individuals were hospitalised and 16 (14%) had bacteraemia. We estimated 900 laboratory-confirmed outbreak cases. Both outbreaks originated from Danish-produced chicken meat. These outbreaks reveal the vulnerabilities in the current prevention and control framework given by the regulatory tolerance for *Campylobacter* in fresh poultry meat.

In July–October 2025 we identified two concurrent *Campylobacter jejuni* outbreaks of cluster type ST49#3 and ST52#4 (Danish nomenclature) through the national whole genome sequencing (WGS) surveillance in Denmark. The two outbreaks were notable in severity and magnitude, and they were traced to Danish chicken meat. We report on the occurrence, progression and characteristics of these outbreaks to shed light on the persistent problem of contaminated chicken meat and its implications for public health.

## Setup and identification of the clusters

In Denmark, around 5,000 laboratory-confirmed *Campylobacter* cases are notified yearly through the national surveillance system with each species-specific infection recorded once per individual in any 6-month period [1].

Since 2019, Statens Serum Institut (SSI) has performed national WGS-based surveillance, covering 10–15% of confirmed *Campylobacter* cases [2]. Through the platform for national sequence-based surveillance of food-borne infections (SOFI), bacterial genomes from human cases are routinely analysed and compared with genomes generated in the Danish Veterinary and Food Administration’s (DVFA) surveillance of food and animal sources.

During summer 2025, ST49#3 and ST52#4 rapidly expanded and were identified countrywide, prompting intensified microbiological and epidemiological investigations. Both clones had been detected in patients and Danish-produced chicken meat in previous years.

Routine surveillance triggers an investigation when ≥ 10 genetically related isolates are detected within 90 days. The threshold was reached for ST49#3 on 2 September (week 36) and for ST52#4 on 12 August (week 33).

## Microbiological and epidemiological profile of the clusters

Between 1 July and 15 October, 2,855 human campylobacteriosis cases were notified in Denmark, with 355 (12%) *Campylobacter* isolates received from Danish clinical microbiology laboratories. Isolates were sequenced and analysed in SOFI, where allele calling was performed using chewBBACA [3] and the 1,343-locus core genome multilocus sequence typing (cgMLST) (Oxford) scheme [4]. Genomes with ≥ 95% loci called were included in the analysis. Species identification, 7-locus MLST typing and antimicrobial resistance prediction were performed in SOFI.

Genetic clusters were defined as groups of isolates clustering with ≤ 5 allelic differences (ADs) using single-linkage clustering. *Campylobacter* isolates originated from all five Danish regions, with routine coverage from four regions and a few additional isolates from the fifth.

To ensure adequate sequencing coverage of national *Campylobacter* cases, clinical microbiology laboratories were requested to temporarily submit additional isolates or samples in September–October 2025, increasing the proportion of sequenced cases and contributing to totally 355 isolates.

Case definition is presented in Box.

Box **Definition of *Campylobacter jejuni* ST49#3 and ST52#4 outbreak cases, Denmark, July–October 2025**

**Outbreak case:**

• *C. jejuni* isolated from a clinical specimen between 1 July and 15 October 2025 regardless of age, sex or travel history

AND

• *C. jejuni* isolate of outbreak clone of ST49#3 within 5 ADs OR ST52#4 within 5 ADs.

AD: allelic difference; cgMLST: core genome MLST; MLST: multilocus sequence typing; ST: multilocus sequence type; #: indicates clusters defined by whole genome sequencing within the ST.

**Figure 1:**
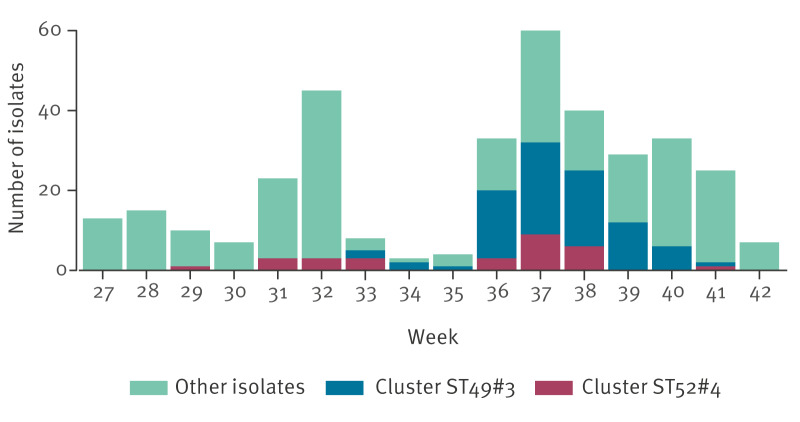
*Campylobacter* isolates from notified cases of campylobacteriosis, by outbreak clone and week of sampling, Denmark, 1 July–15 October 2025 (n = 355)

Overall, 83 isolates were assigned to ST49#3 and 29 to ST52#4. Figure 1 and Table summarise the case characteristics. Both clones were identified nationally, with 106 (95%) of 112 cases domestically acquired. Isolates from both clones carried the *gyrA* T86I mutation and were thus predicted as resistant to ciprofloxacin and nalidixic acid. Sequencing confirmed that these clones matched concurrent isolates from Danish chicken meat identified by DVFA, establishing Danish chicken as the source of each outbreak.

ST: multilocus sequence type; #: indicates clusters defined by whole genome sequencing within the ST.

Number of isolates by outbreak clone: ST49#3 (n = 83); ST52#4 (n = 29); other isolates (n = 243).

**Table:** Characteristics of notified cases with *Campylobacter jejuni* outbreak clones ST49#3 and ST52#4, 1 July–15 October 2025, and notified campylobacteriosis cases in 2024, Denmark

Characteristic	1 July–15 October 2025				2024 (n = 5,547)	
	ST49#3 (n = 83)		ST52#4 (n = 29)			
	N	%	n	%	n	%
Sex						
Female	41	49	14	48	2,548	46
Male	42	51	15	52	2,999	54
Age (years)						
Age range	4–89		2–90		0–98	
Median	57		63		45	
Clinical						
Hospital admission^a^	30	36	15	52	905	16
Positive blood culture	15	18	1	3	59	1
Death^b^	0	0	0	0	12	0

ICD-10: 10th revision of the International Classification of Diseases (https://icd.who.int/browse10/2019/en); ST: multilocus sequence type; #: indicates clusters defined by whole genome sequencing within the ST.

^a^ Hospital admission: any admission lasting ≥ 12 h within ± 7 days of a positive sample and with at least one ICD-10 code possibly related to campylobacteriosis.

^b^ Death: within 15 days of a positive sample.

Patient interviews (n = 35) were conducted using a standardised questionnaire covering food consumption, travel history, and potential exposure sources during the week before symptom onset. All patients reported eating chicken meat.

## Severity and magnitude of the clusters

Hospitalisation data were obtained from the Danish National Patient Registry (LPR) [5] and defined as any admission lasting ≥ 12 h, within ± 7 days of a positive sample and with at least one possibly relevant diagnostic code (ICD-10; 10th revision of the International Classification of Diseases (https://icd.who.int/browse10/2019/en) (Table). Data on positive blood cultures were obtained from the National Register of Enteric Pathogens, maintained by the SSI reference laboratory using information from the Danish Microbiology Database (MiBa) [6]. Mortality data were from the Danish Civil Registration System [7] to identify deaths within 15 days of a positive sample.

**Figure 2:**
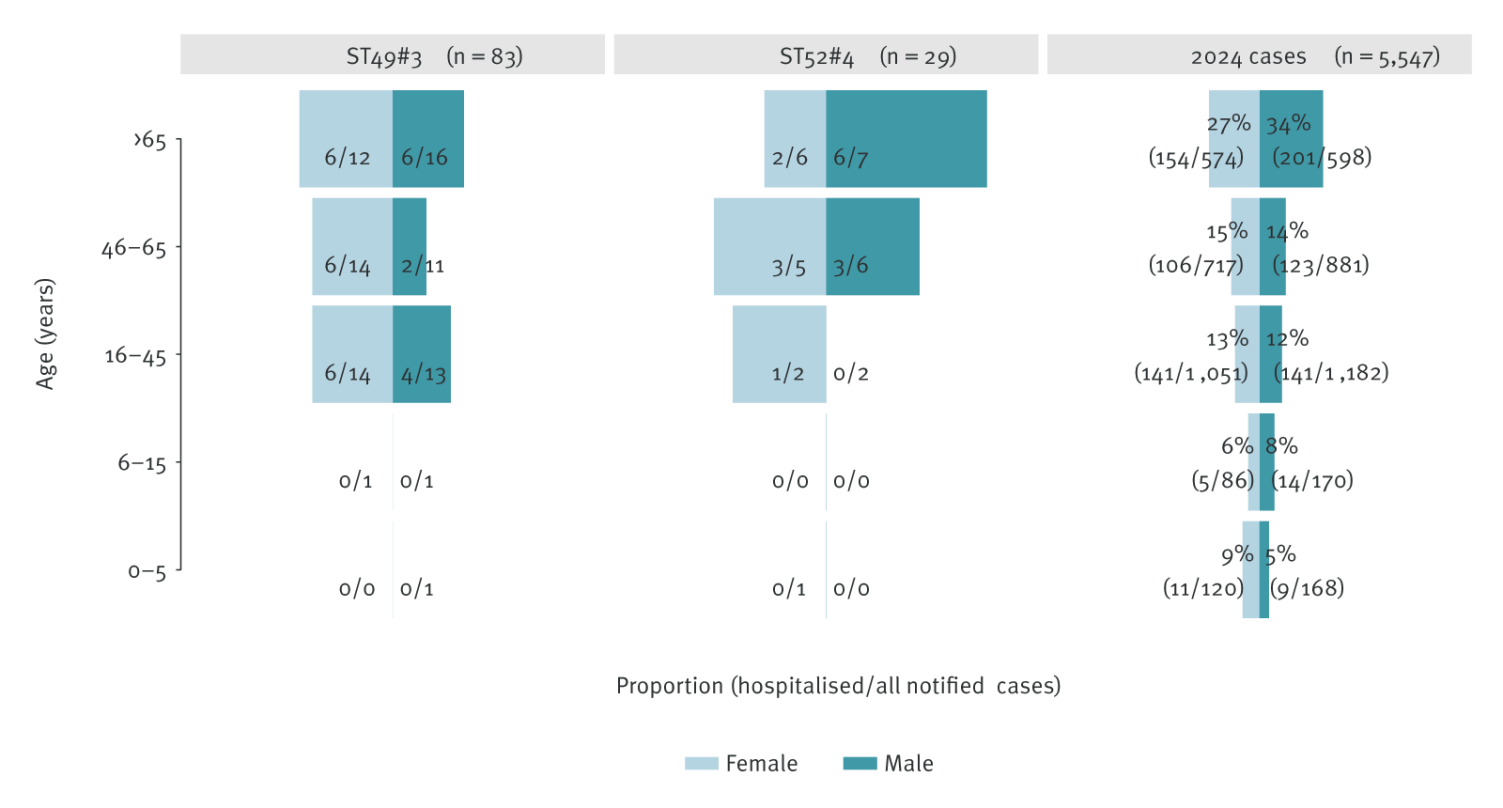
Hospitalisation of cases with *Campylobacter jejuni* outbreak clones ST49#3 and ST52#4, 1 July–15 October 2025, and notified campylobacteriosis cases in 2024, by age and sex, Denmark

**Figure 3:**
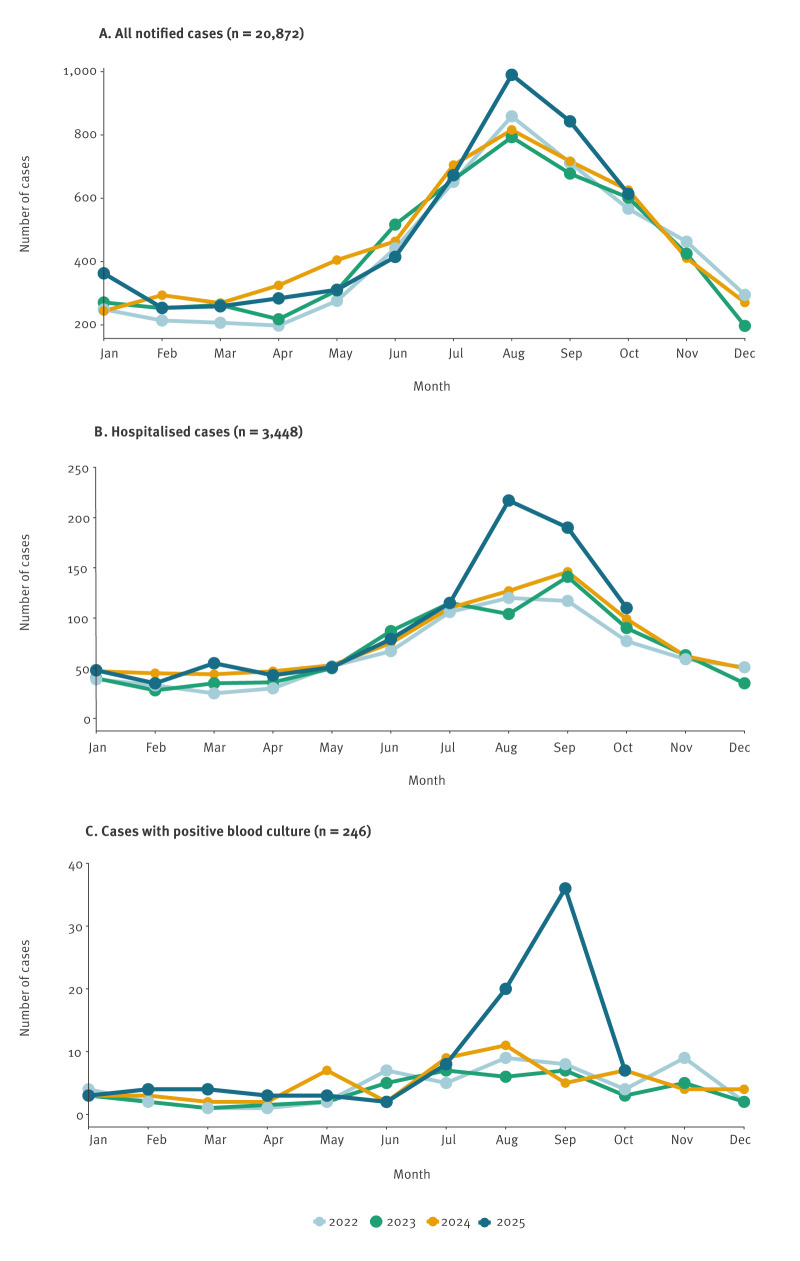
Cases of campylobacteriosis, by month of sampling, Denmark, 2022–2025^a^

^a^ Data on 2025 cases cover January–October.

Thirty (36%) of 83 ST49#3 cases and 15 of 29 ST52#4 cases were hospitalised. Fifteen (18%) ST49#3 cases and one ST52#4 case had a positive blood culture. No deaths were recorded. Table and Figures 2–3 summarise case characteristics and clinical severity.

ST: multilocus sequence type; #: indicates clusters defined by whole genome sequencing within the ST.

Numbers of all cases are presented in the labels above the figures. Numbers of hospitalised cases: ST49#3 (n = 30); ST52#4 (n = 15); 2024 cases (n = 905).

Panel A. Case numbers: 2022 (n = 5,134); 2023 (n = 5,185); 2024 (n = 5,547); 2025 (n = 5,006).

Panel B: Case numbers: 2022 (n = 776); 2023 (n = 824); 2024 (n = 905); 2025 (n = 943).

Panel C. Case numbers: 2022 (n = 54); 2023 (n = 43); 2024 (n = 59); 2025 (n = 90).

Hospital admission: any admission lasting ≥ 12 h within ± 7 days of a positive sample and with at least one ICD-10 code possibly related to campylobacteriosis.

Using a probability-based method routinely applied in our surveillance [8], we estimated the real number of laboratory-confirmed cases for the ST49#3 and ST52#4 outbreaks to be 670 (90% uncertainty interval (UI): 569–779) and 238 (90% UI: 175–312), respectively.

## Discussion

The concurrent outbreaks caused by ST49#3 and ST52#4 were remarkable both in scale and severity. Although both clones were linked to Danish chicken meat by WGS-based comparison, the evidence suggested two separate events. A higher proportion of cases were hospitalised (30/83 and 15/29), compared with 16% nationally in 2024 and 10.8% in an earlier Danish study [9]. Fifteen (18%) ST49#3 cases and one ST52#4 case had bacteraemia, exceeding the Danish baseline of < 0.5% [10]. The markedly higher proportion of bacteraemia suggests potential clone-specific factors enhancing invasiveness. These proportions indicate the highest clinical severity so far recorded for *Campylobacter* outbreaks in Denmark and challenge the perception of campylobacteriosis as a generally mild, self-limiting disease. Outbreaks of comparable magnitude are rare in Denmark: the most recent occurred in 2019, when ST122#1 caused an estimated 700 infections linked to Danish chicken meat [2]. Although *Campylobacter* is frequently detected in poultry production and outbreaks are identified every year, such large and severe events are unusual. The estimated 900 laboratory-confirmed infections — without considering the undiagnosed cases — illustrates the substantial public health impact and highlights how fluctuations in contamination levels or specific clone dynamics can amplify the national disease burden.

The median age of the outbreak cases was higher than of cases detected in 2024, but hospitalised cases were aged 16 to ≥ 65 years, suggesting that age alone could not explain the severity. Possible contributing factors could include higher exposure doses through contaminated products, clone-specific virulence, and/or broader dissemination within the food chain. Regardless of the mechanism, these findings indicate that some *C. jejuni* lineages (e.g. ST49#3) may pose greater risks to humans. Although some genotypes have been associated with invasive disease [11,12], lineage-specific differences are not yet systematically integrated into surveillance or risk assessment.

From a public health perspective, the occurrence of two unusually severe and large outbreaks within a short period reveals the vulnerability of the current *Campylobacter* prevention and control framework for poultry. Both clones were detected in humans and Danish-produced chicken meat, showing that substantial disease events can occur within a regulatory system that is built around process hygiene criteria [13]. In the absence of legal prohibition against *Campylobacter* in fresh, frozen or marinated chicken meat, this framework places much of the practical burden of *Campylobacter* risk reduction on consumers through safe food handling practices. The described outbreaks reveal a misalignment between surveillance and current regulation targets.

## Conclusion

In conclusion, we present evidence of two unusually large, concurrent outbreaks with high levels of hospitalisation and invasive infections. Sequencing was instrumental in revealing these outbreaks and establishing their link to chicken meat, demonstrating that severe clinical disease can occur despite the routine monitoring and control measures currently in place in poultry production. These findings highlight the importance of continued genomic surveillance and coordinated cross-sector investigations to better detect and understand such events.

## Data Availability

Sequencing data from the human isolates from surveillance for 2025 are deposited in the National Centre for Biotechnology Information (NCBI) Sequence Read Archive under BioProject accession number PRJNA1363081 (https://www.ncbi.nlm.nih.gov). As the data form part of an ongoing outbreak investigation they are not yet made publicly available. The data will be released once the investigation is concluded and all 2025 data are cleared for public dissemination.
